# Carboxymethylated and Sulfated Furcellaran from *Furcellaria lumbricalis* and Its Immobilization on PLA Scaffolds

**DOI:** 10.3390/polym16050720

**Published:** 2024-03-06

**Authors:** Kateřina Štěpánková, Kadir Ozaltin, Petr Sáha, Elif Vargun, Eva Domincová-Bergerová, Alenka Vesel, Miran Mozetič, Marian Lehocký

**Affiliations:** 1 Centre of Polymer Systems, University Institute, Tomas Bata University in Zlín, Trida Tomase Bati 5678, 760 01 Zlin, Czech Republic; ozaltin@utb.cz (K.O.); saha@utb.cz (P.S.); domincova_bergerova@utb.cz (E.D.-B.); 2 Department of Chemistry, Mugla Sitki Kocman University, Kotekli, 48000 Mugla, Turkey; vargun@utb.cz; 3 Department of Surface Engineering, Jozef Stefan Institute, Jamova cesta 39, 1000 Ljubljana, Slovenia; alenka.vesel@ijs.si (A.V.); miran.mozetic@ijs.si (M.M.)

**Keywords:** furcellaran, seaweed polysaccharide, sulfation, carboxymethylation, plasma treatment, PLA, scaffolds

## Abstract

This study involved the creation of highly porous PLA scaffolds through the porogen/leaching method, utilizing polyethylene glycol as a porogen with a 75% mass ratio. The outcome achieved a highly interconnected porous structure with a thickness of 25 μm. To activate the scaffold’s surface and improve its hydrophilicity, radiofrequency (RF) air plasma treatment was employed. Subsequently, furcellaran subjected to sulfation or carboxymethylation was deposited onto the RF plasma treated surfaces with the intention of improving bioactivity. Surface roughness and water wettability experienced enhancement following the surface modification. The incorporation of sulfate/carboxymethyl group (DS = 0.8; 0.3, respectively) is confirmed by elemental analysis and FT-IR. Successful functionalization of PLA scaffolds was validated by SEM and XPS analysis, showing changes in topography and increases in characteristic elements (N, S, Na) for sulfated (SF) and carboxymethylated (CMF). Cytocompatibility was evaluated by using mouse embryonic fibroblast cells (NIH/3T3).

## 1. Introduction

Synthetic biopolymers play an important role in effectively substituting damaged tissue, applicable for both in vivo and in vitro scenarios [1]. Scaffolds provide a structurally crucial three-dimensional (3D) environment that defines the shape of tissue regeneration. They should exhibit essential characteristics, including a highly interconnected porous structure enabling cell penetration and nutrient transport, biocompatibility, biodegradability, appropriate mechanical properties, and surface modifications with suitable topography to promote cell adhesion and growth [2,3]. Also, their degradation rate should align with the pace of tissue growth [4]. Therefore, the existing challenge in tissue engineering revolves around creating scaffolds with ideal physical and biological properties that foster efficient cell growth, all the while preserving appropriate mechanical characteristics for the in vivo environment.

Among all sustainable polymer materials, polylactide (PLA) emerges as a promising material for scaffold construction [5,6]. This biopolymer, sanctioned for direct contact with biological fluids by the Food and Drug Administration (FDA), boasts non-toxic hydrolysis by-products and possesses sufficient mechanical stability [7]. Various methods are available for producing a porous PLA matrix customized for specific applications, such as tissue engineering, drug loading, or wound dressing [8,9]. These techniques encompass approaches such as solvent casting/porogen leaching, freeze-drying, electrospinning, or material extrusion [10,11]. Porogen leaching emerges as a straightforward and exceedingly efficacious technique for crafting porous scaffolds, granting direct command over both the size and dispersion of the pores [12]. The determining factor for porosity lies in the ratio of porogen to polymer, while the size of the porogen dictates the final scaffold’s pore size. Controlling the porosity and pore size holds significance as it affects drug release, oxygen and water vapor transmission of wound dressings [13,14]. The optimal porosity and pore size of porous films depends on the wound type and their intended application. Moreover, porous architecture of PLA films should exhibit large surface are to volume ratio, suitable pore interconnectivity, and the ability to conform to the shape of the wound [15].

Nevertheless, PLA harbors certain limitations that curtail its suitability in the biomedical realm. Notably, the existence of pendant methyl groups and the lack of reactive functional groups in its structure bestow a relatively hydrophobic surface with low surface energy upon PLA. This characteristic impedes the effective attachment and proliferation of cells [16]. Numerous techniques for surface functionalization have been employed to augment the bioactivity of PLA scaffolds. These include physical methods like plasma treatment, chemical processes such as aminolysis, and biological approaches like the coating and immobilization of biologically active molecules [17,18,19]. In comparison to alternative methods, plasma technology offers distinct advantages, such as the utilization of non-toxic chemicals, processing devoid of heat, and modification without altering the bulk properties. These approaches result in the formation of functional groups such as NH_x_ or C=O, COOH, and OH functional groups on the surface of the scaffold, enabling subsequent covalent or physical bonding with various species, including proteins, peptides, and polysaccharides [20]. Physical bonding (physisorption) of a biomolecule onto a polymer surface can occur through van der Waals forces, hydrogen bonds, and electrostatic interactions [21]. However, the impermanence of physical adsorption might be a limitation in terms of the stability of immobilized biomolecules after rinsing with polar solvents [22]. Therefore, the formation of strong polar interactions and hydrogen bonding between the biomolecule and the pre-treated surface may thereby increase the efficiency of immobilization when combined with plasma treatment [23,24]. Furthermore, attaining a regulated release of bioactive molecules can enhance clinical effectiveness. It is advantageous for tissue engineering scaffolds to serve not only as 3D matrices but also as delivery vehicles for bioactive molecules, thereby boosting cellular activity during the process of tissue repair [25].

Among suitable bioactive molecules, anionic polysaccharides analogous to glycosaminoglycans (GAGs) are widely employed for the immobilization of biomaterials, given their ability to support cell proliferation, differentiation and cell adhesion [26]. Furcellaran, derived from the red algae (*Furcellaria lumbricalis*), is a naturally sulfated anionic galactan. It exhibits both structural and functional resemblances to κ-carrageenan, albeit differing in the quantity of sulfate esters. Its composition consists of a recurring backbone with alternating (1 → 4)-3,6-anhydro-α-d-galactopyranose-(1 → 3)-β-d-galactopyranose-4′-sulfate structural units, featuring approximately one sulfate ester per every three monomer residues [27]. In recent studies, it has been observed to enhance the proliferation of embryonic stem cells and demonstrate anticoagulant properties [28,29]. Numerous studies indicate that the inherent bioactivities of native polysaccharides are relatively low but can be significantly improved through molecular modifications [30]. Sulfation or carboxymethylation emerges as a viable approach to address the limited water solubility and amplify the biological activity of natural polysaccharides through the alteration of their structural and conformational characteristics [31,32,33,34].

**Figure 1:**
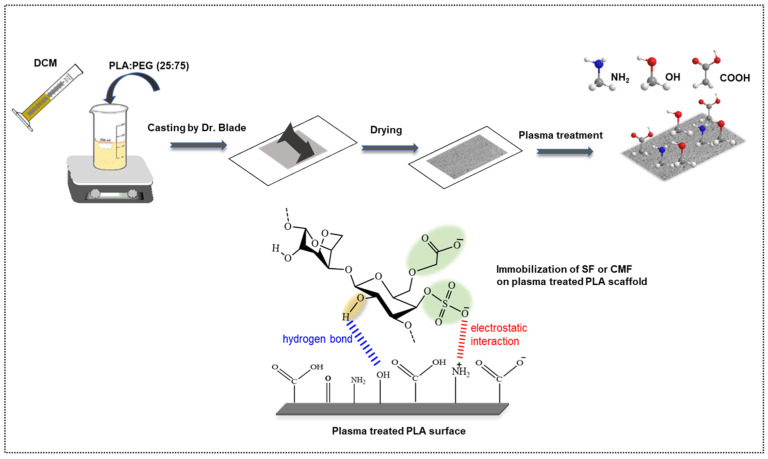
Schematic representation of PLA scaffold fabrication and its surface functionalization with furcellaran derivates via RF plasma.

In our previous study, we explored the bioactive potential of furcellaran by depositing it on PET film [28]. In this study, we present a novel approach where furcellaran is carboxymethylated and compared to sulfated furcellaran, aiming to assess their respective effects on the surface functionalization of a PLA scaffold. Toward developing a highly porous PLA scaffolds (Figure 1), polyethylene glycol (PEG) was used as a forming agent at a mass ratio of 75%. The thickness of the PLA scaffold was adjusted to 25 μm, aiming to enhance its in vivo degradation kinetics following the release of furcellaran [35]. To activate the surface of the PLA scaffold and prepare it for subsequent interaction with biomolecules, RF air plasma was employed. The resulting PLA-FUR, PLA-SF, and PLA-CMF variants were prepared by immobilization of a pyridinium salt form of native furcellaran (FUR), sulfated furcellaran (SF), and carboxymethyl furcellaran (CMF) on the RF plasma treated surface via physisorption. The impact of surface functionalization was evaluated based on the physicochemical properties of the PLA scaffold surface, as well as its influence on the cellular response of mouse embryonic fibroblast cells.

## 2. Materials and Methods

### 2.1. Materials and Reagents

Pellets of Poly(lactic acid) (PLA) 4032 D were acquired from Nature Works (Blair, NE, USA). Furcellaran (FUR) [*M_w_* 2.55 × 10^5^ Da; Estgel 1000] was sourced from Est-agar a.s. (Kärla village, Estonia). Amberlite^®^ IR-120, H^+^ ion-exchange resin (Sigma-Aldrich, St. Louis, MO, USA), Dialysis tubing (MWCO—12,000 Da), Pyridine 99.9%, Dimethyl Sulfoxide (DMSO) 99.9%, and Sulfur trioxide pyridine complex (SO_3_∙Py) were acquired from Sigma-Aldrich (St. Louis, MO, USA). Chloroacetic acid (MCA) 99% (Thermo Scientific, Waltham, MA, USA), 2-propanol, and hydrochloric acid (HCl), 37% (*v*/*v*) (VWR chemicals, Rosny-sous-Bois, France) were employed in the carboxymethylation of furcellaran.

### 2.2. Sulfation of Furcellaran

**Figure 2:**
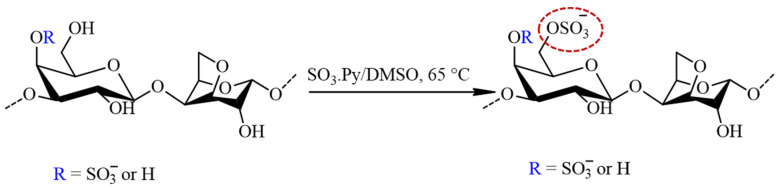
The schematic representation of the sulfation process for furcellaran with SO_3_∙Py complex results in a sulfate group being incorporated to the G-6 position (circled in red).

The sulfation of furcellaran was preceded by the preparation of FUR in a pyridinium salt form by cation exchange chromatography. The eluate was neutralized with pyridine, dialyzed (MWCO 12,000) against demineralized water and the solution was freeze dried for 48 h. The FUR (pyridinium salt form) was solubilized in anhydrous DMSO (30 mL) by agitating for 30 min at ambient temperature. Afterwards, 4:1 molar ratio of SO_3_∙Py complex to sugar residues were added and the mixture was stirred for 3 h at 65 °C (Figure 2). The reaction mixture was cooled to laboratory temperature, dissolved in 1 M NaOH and precipitated in graded ethanol. Finally, sulfated furcellaran (SF) was dialyzed for 3 days against DEMI water and lyophilized.

### 2.3. Carboxymethylation of Furcellaran

**Figure 3:**
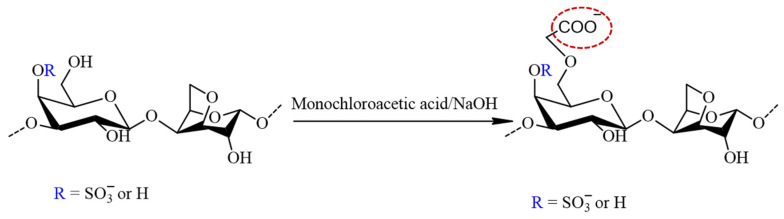
The reaction scheme of the furcellaran carboxymethylation with MCA in alkalic media. The structure represents the target carboxymethyl incorporated to the G-6 position (circled in red) and does not reflect the strict composition of the sample.

The carboxymethylation of furcellaran (Figure 3) followed Williamson’s ether synthesis for alkaline activation. Initially, 200 mg of FUR (pyridinium salt form) was suspended in 40 mL of an 80% isopropanol aqueous solution. To form alkoxy-furcellaran, 4 mL of 20% NaOH solution was added dropwise with stirring, and the reaction mixture was heated at 35 ± 5 °C for 1 h. Monochloroacetic acid (MCA) (800 mg) was dissolved in 4 mL of 20% NaOH aqueous solution by stirring at room temperature until complete dissolution was achieved. The MCA solution was added dropwise with a syringe to the reaction mixture, and the temperature was maintained at 55 ± 1 °C for 5 h under stirring. After cooling the solution to room temperature, 0.5 mol/L of HCl was added to adjust the pH to 7–8. The solution of carboxymethyl furcellaran (CMFUR) was concentrated, dialyzed, and lyophilized.

### 2.4. Characterization of Sulfated Furcellaran Carboxymethyl Furcellaran

For structural analysis of the native FUR, SF and CMF, the infrared spectrometer (Nicolet iS5, Thermo Scientific, Grand Island, NY, USA) equipped with the germanium crystal in the ATR mode in a range of wavelengths 4000–600 cm^−1^ was used.

For elemental composition, an elemental analyzer (FLASH 2000 CHNS/O + MAS200R, Thermo Fisher Scientific, Sunnyvale, CA, USA) was employed. The detection limit in FLASH analysis is below 0.5–1%.

### 2.5. Degree of Substitution of Sulfated Furcellaran and Carboxymethyl Furcellaran

The degree of substitution (DS) of SF was determined using the percentages of sulfur (% S) and carbon (% C), as follows:

(1) $$DS=2.25\times \frac{S\%}{C\%}$$

Carboxymethyl content was assessed using the method of neutralization titration [32]. The determination of the carboxymethyl group (C) content was carried out through the following procedure:

(2) $$C=\frac{\left[V_{0}M_{0}-\left(V_{2}-V_{1}\right)M\right]}{W}$$

Here, V_0_ represents the equivalent volume of NaOH added (in mL), V_2_ stands for the equivalent volume of HCl added (in mL), V_1_ denotes the volume of hydrochloric acid used for titration of the sample (in mL), M_0_ represents the NaOH concentration used (0.5 mol L^−1^), M is the concentration of HCl used for titration (0.1 mol L^−1^), and W is the mass of the sample (in g). The calculation of DS is determined as follows:

(3) $$DS=\frac{0.162C}{\left(1-0.058C\right)}$$

### 2.6. Preparation of PLA Scaffolds

The fabrication of the PLA scaffold was carried out using the solution casting/porogen leaching technique as per the method described by Ozaltin et al. [35]. During this procedure, a blend of PLA and PEG (25:75) was dissolved in dichloromethane at a concentration of 143 g/L. The resultant solution was then applied to a clean glass plate using the doctor-blade method, forming PLA/PEG films with a thickness of 25 µm. Following this, the films were submerged in DEMI water for 48 h at ambient temperature to extract PEG, and subsequently, they were dried in an oven for 12 h at 50 °C, resulting in the production of a highly porous PLA scaffold.

### 2.7. Surface-Functionalization of PLA Scaffolds

The PLA scaffold surfaces underwent plasma treatment (Diener Electronic, Nagold, Germany) for a duration of 60 s at 13.56 MHz. The discharge matching power was configured at 50 W with the air feed rate 20 sccm. The chamber pressure was approximately 60 Pa. For FUR, SF, CMF immobilization, the plasma treated scaffolds were immersed in 0.2% (*w*/*v*) polysaccharides acidified aqueous solution (0.5 M HCl; pH 5) for 24 h at ambient temperature under agitation. Afterwards, the scaffolds were taken out of the solution, gently rinsed by PBS and left dried for 3 h at 35 °C in oven. The resulting PLA-FUR, PLA-SF and PLA-CMF were characterized.

### 2.8. Surface Characterization of Treated PLA Scaffolds

For the examination of alterations in the chemical composition of furcellaran surfaces and the chemical binding state, X-ray Photoelectron Spectroscopy (XPS) utilizing TFA (Physical Electronics, Chanhassen, MN, USA) with MultiPak v7.3.1 software for element concentration determination was employed. The samples were exposed to X-rays with a 400 μm spot size generated using monochromatic Al Kα1,2 radiation at 1486.6 eV. The emitted photoelectrons were captured by a hemispherical analyzer positioned at a 45° angle relative to the normal plane of the sample surface.

To assess the impact of surface functionalization by FUR, SF, and CMF on PLA surface wettability, the water sessile drop contact angle method was utilized through a SEE system (by Advex Instruments, Brno, Czech Republic). Droplets of the testing liquid (5 µL) were deposited onto the sample surface and recorded by CCD camera system.

The surface morphology of PLA, PLA-SF, and PLA-CMF scaffold was visualized using a NANOSEM 450 (FEI, Thermo Fisher Scientific, Hillsboro, OR, USA) scanning electron microscope with an accelerating voltage of 5 kV. Scaffolds were coated with Au/Pt and images were at a magnification of 20,000×.

### 2.9. Cytotoxicity

The cytotoxicity was evaluated on mouse embryonic fibroblast cell line (NIH/3T3, ATCC^®^ CRL-1658^TM^, Manassas, VA, USA) following the EN ISO 10993-5 [36]. The samples (10 × 10 mm) were sterilized with UV radiation (λ = 253.7 nm; 30 min). As a culture medium, ATCC-formulated Dulbecco’s modified Eagle’s medium (10% calf serum and 100 U mL^−1^ penicillin/streptomycin (BioSera, Nuaille, France)) was used. The cells were seeded onto samples (2 × 10^4^ cells per mm^2^) and incubated at 37 ± 1 °C for 72 h. The cell viability was assessed using the 3-(4,5-dimethylthiazol-2-yl)-2,5-diphenyltetrazolium bromide (MTT) assay (Duchefa Biochemie, Amsterdam, The Netherlands). After the removal of the culture medium, the cells were supplemented with 100 µL of growth media containing 5 mg/mL of MTT staining solution in PBS. Afterwards, the cells were cultured for 4 h at 37 °C. After the growth medium was removed, the formed formazan crystals on the sample surface were removed by DMSO. The absorbance was recorded at λ = 570 nm (test) and 690 nm (reference).

## 3. Results

### 3.1. Chemical Structure of Furcellaran and Its Derivates

**Figure 4:**
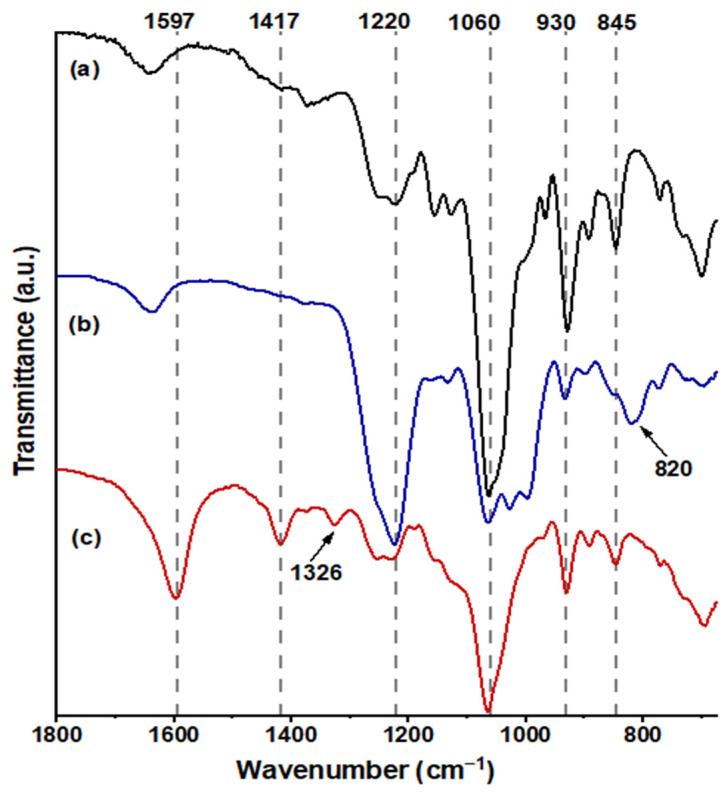
FT-IR spectra obtained from the samples are as follows: spectrum (**a**) corresponds to FUR, spectrum (**b**) to SF, and spectrum (**c**) to CMF in the range 1800–690 cm^−1^.

In Figure 4a, FUR exhibited distinctive bands at 845 cm^−1^ (C–O–S stretching), indicative of the axial galactose-4-sulfate; 1060 cm^−1^ (CH–O–CH_2_ stretching); 930 cm^−1^, suggesting the presence of the 3,6-anhydro-galactosidic bond; and 1220 cm^−1^ (S=O stretching), signifying the total sulfate content [37]. Following sulfation (Figure 4b), a novel absorption band emerged at 820 cm^−1^ (C–O–S stretching), indicating galactose-6-sulfate, and the intensity of the absorption band at 1220 cm^−1^ markedly increased [38]. The confirmation of carboxymethylated FUR, as depicted in Figure 4c, was validated by the emergence of a band at 1597 cm^−1^ (–COO−), associated with the stretching vibration of the asymmetric carboxyl group, a band at 1417 cm^−1^ related to the symmetric C–O stretching of the carboxyl group, and a scissoring vibration of the methylene group at 1329 cm^−1^ [34,39].

### 3.2. Elemental Composition

Table 1 shows the analyzed values of nitrogen, carbon, hydrogen, and sulfur in mass percentages (wt%). The sulfur content detected in FUR was 2.0%, but it increased to 8.0% in SF and the DS was calculated to be 0.8. The increased sulfate content of SF as compared to FUR indicates successful sulfation. These findings are in accordance with Gunasekaran et al. [40] and Dhahri et al. [41]. Moreover, the elevation in nitrogen content is probably an outcome of heterogeneous sulfation in pyridine, which may include nitrogen-containing impurities arising from side reactions with pyridine [42]. % Carbon was found to decrease in SF. The incorporation of sulfate group introduced sulfur and oxygen without proportional increase in carbon, resulting in a lower carbon-to-mass ratio.

This aligns with findings from related studies [43,44]. A significant reduction in sulfur content indicates de-sulfation in CMF, as the highly alkaline conditions in the reaction induced hydrolysis of sulfate in C-4 of the -d-galactose unit, potentially leading to intramolecular nucleophilic displacement [45]. These data are consistent with FTIR analysis. Both samples, CMF and FUR, exhibit similar amounts of nitrogen (approximately 0.1%) and hydrogen (approximately 4.5–5%). The presence of nitrogen traces in FUR and CMF can be ascribed to protein contaminants, which should be effectively eliminated from the cell wall during the extraction [46]. This is due to the fact that proteins are integral components of the cell wall structure and are closely linked with polysaccharide [47]. Furthermore, there is a slight increase in the carbon content (by approximately 2–3%) in the CMF sample, indicating successful attachment of the −CH_2_COONa group and the DS was calculated to be 0.3. The increase in oxygen content indicate carboxymethyl group incorporation into polysaccharide structure as well and is directly proportional to the extent of treatment [48,49]. While the employed methods did not detect sodium, its expected presence introduces some uncertainty when determining oxygen content through the difference in elemental analysis [50].

### 3.3. Surface Wettability Analysis

**Figure 5:**
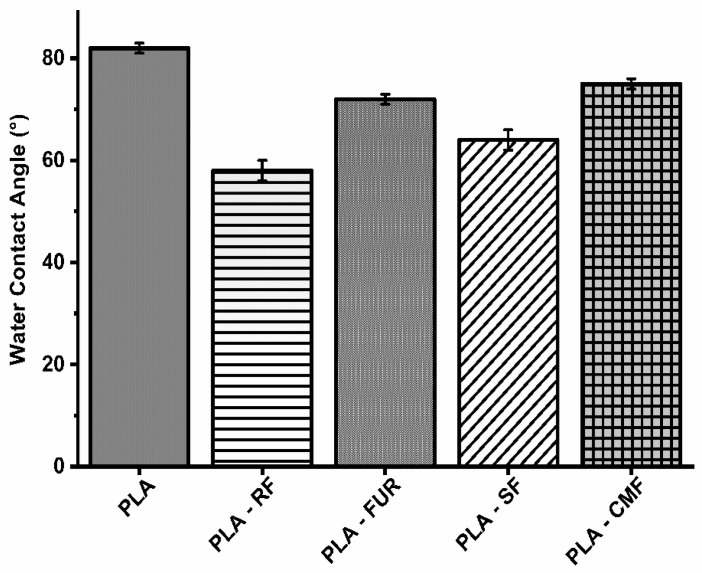
Water contact angles on surface of scaffolds.

The impact of surface modification on hydrophilicity was assessed using static contact angle (WCA) measurements (Figure 5). The RF oxygen plasma treatment resulted in a significant reduction in the water contact angle on the PLA porous surface (58.2°), attributed to an increase in surface area and the presence of oxygen-containing bonds [51,52]. The enhanced hydrophilic nature of plasma treated PLA surface results in higher amount of subsequently adsorbed polysaccharide. Surface functionalization by FUR, SF, and CMF did affect hydrophilicity of PLA scaffolds with slight increase of WCA (72.1°–75.1°) compared to oxygen plasma treated surface, but WCA was lower than on unfunctionalized PLA scaffold. Furcellaran is recognized for displaying solubility traits characteristic of hydrophilic colloids, facilitated by sulfates and hydroxyls in their backbone, whereas 3,6-anhydro-d-galactose residues (3,6-AG) are comparatively more hydrophobic [53]. The degree of substitution (DS) is associated with an enhancement in the hydrophilic characteristics of carboxymethylated and sulfated polysaccharides. The surface of PLA treated with SF demonstrated greater hydrophilicity in comparison to that treated with CMF. A lower DS observed in CMF along with decrease in sulfate content stemming from the higher NaOH concentration, is reflected in constrained incorporation of hydrophilic groups [54]. Moreover, the solubility and ensuing hydrophilicity are profoundly affected by the pyridinium salt form of FUR.

### 3.4. Surface Elemental Analysis of PLA Scaffolds

The chemical composition of PLA scaffold surfaces before and after SF and CMF functionalization was clearly demonstrated through XPS analysis. Table 2 displays the atomic concentration of specific elements of interest—C, O, N, S for pristine PLA scaffold as a reference—PLA surface treated with sulfated furcellaran (PLA-SF), and PLA surface treated with carboxymethylated furcellaran (PLA-CMF). The untreated PLA scaffold contained 62.8% carbon atoms and 37.2% oxygen atoms, which is in agreement with theoretical expectations derived from the chemical formula of PLA. Confirmation of surface functionalization by SF and CMF was established through an increase in the percentage of sulfur and nitrogen on the surfaces of PLA-SF and PLA-CMF. Oxygen concentration of PLA-CMF was slightly higher compared to PLA-SF scaffolds, indicating the presence of more carboxymethyl groups [39]. The higher nitrogen content (1.4%) in PLA-SF compared to PLA-CMF may stem from both plasma treatment and pyridine originated from the sulfation. Since nitrogen (1.1%) from the plasma treatment was still detectable, it can indicate that SF and CMF was adsorbed onto PLA surface with thickness < 10 nm (XPS detection limit).

**Figure 6:**
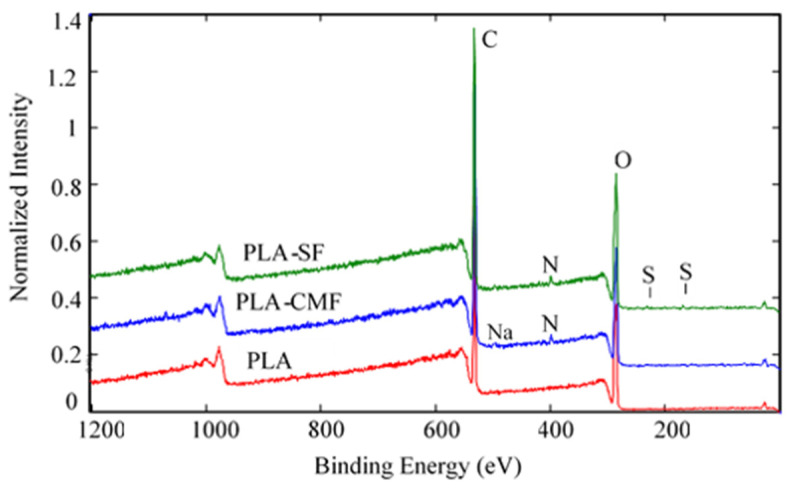
Full survey-scan spectra of PLA-SF, PLA-CMF, and pristine PLA.

**Figure 7:**
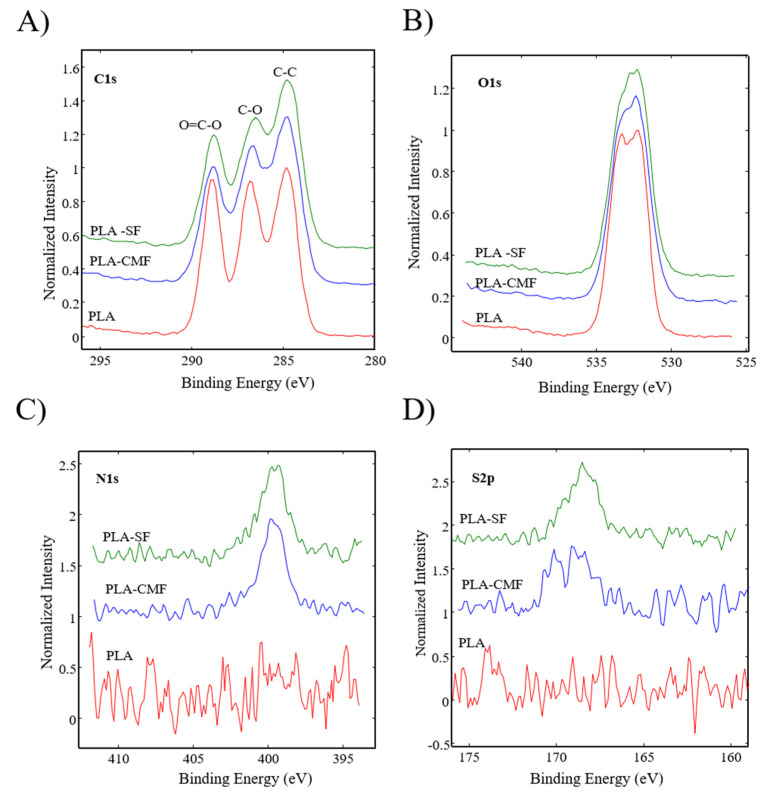
High-resolution spectra for (**A**) carbon C(1 s), (**B**) oxygen O(1 s), (**C**) nitrogen N(1 s), and (**D**) sulfur S(2p) are depicted for all three samples.

Figure 6 illustrates the XPS spectra of the samples along with their surface elemental compositions. The sharp peak at ~285 eV corresponds to C(1 s), and the peak at ~533 eV (Figure 7B) corresponds to O(1 s), reflecting the chemical structure of PLA. As observed in Figure 7C), the surfaces of functionalized PLA scaffolds also exhibit nitrogen N(1 s) assigned to N–H at ~400 eV due to air plasma treatment, which is composed of oxygen and nitrogen radicals.

Following surface functionalization (Figure 7D), both PLA-SF and PLA-CMF samples revealed a sulfur peak S2p3/2 at ~167.7 eV for the sulfate group, indicating the presence of furcellaran on the PLA scaffold surface. Additionally, the deposition of CMF displayed a small peak corresponding to the contribution of Na(1 s), assigned to the −CH_2_COONa group (Figure 6). The inset shows the deconvolution of the C1s peak into three components (Figure 7A), located at ~284.6 eV (C–C/C–H), ~286.3 eV (C–O), and ~289.0 eV (O–C=O). In the C1s peak decomposition, the absence of C-N bonds could be attributed to the relatively low nitrogen content compared to oxygen, primarily arising from PLA ester bonds. No significant shift in the binding energies for C1s and O1s was observed across the samples. A minor decline in the C–O and COO– peaks for PLA-SF and PLA-CMF suggests the bonding of furcellaran derivates onto plasma-treated surface [55]. 

### 3.5. Surface Morphology and Characterization of PLA Scaffolds

**Figure 8:**
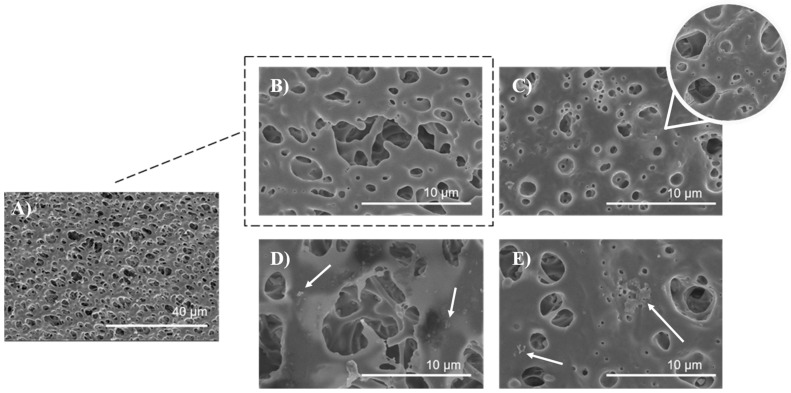
SEM images of PLA scaffolds (**A**) untreated at magnification of 2500×, (**B**) untreated PLA at magnification of 20,000×, (**C**) plasma treated, (**D**) SF functionalized, (**E**) CMF functionalized, all at magnification of 20,000×.

The surface morphology of the untreated, air plasma-treated, and polysaccharide-functionalized porous PLA scaffold was examined using SEM. The SEM image of untreated PLA (Figure 8A,B) displayed a smooth and uniform surface with a highly interconnected pore structure. In contrast, low-pressure plasma treatment resulted in an increase in surface roughness and the formation of micropittings due to oxidative products caused by polymer chain scission, etching, and degradation processes (Figure 8C) which has been observed in other studies as well [35,56]. Plasmating the surface changed the topographic properties, wettability, and increase surface area, resulting in more favorable adsorption of furcellaran derivated due to mechanical interlocking [21]. This phenomena is in a good agreement with other studies [18,19]. Additionally, a three-dimensional model defined by geometry, which closely mimics the extracellular matrix (ECM) microenvironment is crucial for cell adhesion, proliferation, differentiation, mechano-responses, and cell survival [57]. The scaffold has a total thickness of 25 mm, with pore sizes ranging from 500 nm to 10 μm [35]. As seen in Figure 8D), the distribution of SF was more likely homogenous with some flocculated particles on the polymer surface (indicated by a white arrow), as well as inside of pores, indicating a complex adsorption behavior that extends beyond the surface. Visible particles of polysaccharide gather in specific regions of the polymer surface due to a combination of higher affinity, charge distribution, concentration of polysaccharide solution and electrostatic interactions [18,21]. In the case of PLA-CMF scaffold (Figure 8E), scaffold surface is similar like PLA_SF surface, but in addition to that, few aggregated particles were observed on the surface (indicated by a white arrow) which may be due to uneven distribution of the deposited polysaccharide and its unsuitable local electrostatic binding with the polymer surface.

### 3.6. In Vitro Fibroblast Cytocompatibility

**Figure 9:**
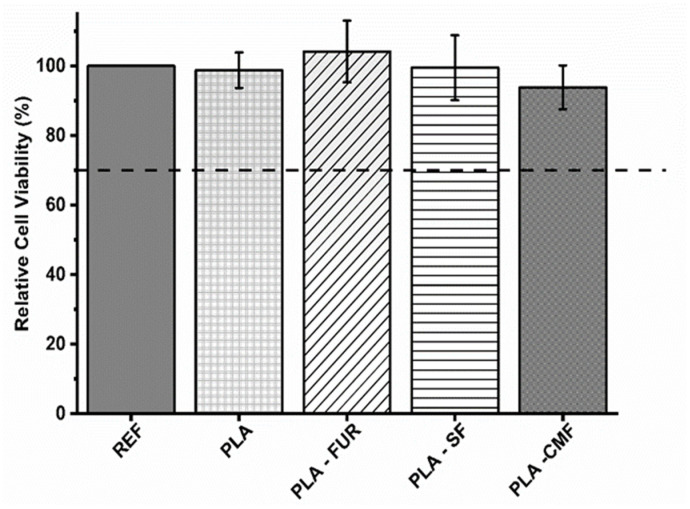
Relative cell viability values as % of the expanded polystyrene as a reference. The values are presented in triplicate.

To assess potential cytotoxic effects of untreated and functionalized PLA scaffolds on expanded NIH/3T3 cells, a direct contact assay was conducted. The results are presented in triplicate with a negative control of polystyrene utilized for comparison. As presented in Figure 9, untreated PLA scaffold show biocompatibility despite its hydrophobic nature, low surface energy, and absence of reactive functional groups. The PLA scaffold was not cytotoxic also in the study of Biagini et al. [58] for hASCs and expanded CD133^+^ cells. Other investigations have demonstrated that PLA scaffolds enhance the proliferation of fibroblasts [59]. The pore size and interconnectivity of the pores may be an important factor influencing cell responses by promoting oxygen diffusion and cell proliferation [5,60,61]. In addition to the material’s surface chemistry and surface energy, its biocompatibility is profoundly affected by surface topography [62]. Micro/nano-scale roughness and distinct patterns play a crucial role in controlling cell attachment and proliferation, impacting the size, shape, and also spatial distribution of adhered cells [63]. As it was previously mentioned, plasma treatment resulted in an increase of surface roughness which is favorable for cell-material surface interactions. However, the efficacy of plasma treatment lies in its ability to modify the surface of the PLA scaffold without altering the scaffold’s interior, leading to localized non-uniformity in plasma treatment and possible inhomogeneous distribution of furcellaran inside of pores [64]. Among all of the functionalized PLA surfaces, native FUR shows the highest biocompatibility. The non-cytotoxic characteristics of native furcellaran align with our earlier investigation [28], wherein furcellaran deposition onto polyethylene terephthalate (PET) surfaces demonstrated cytocompatibility. The PLA-SF sample demonstrates a favorable effect as well, although it does not exceed the cell viability of native furcellaran. One study demonstrated, that the decrease in cell viability is attributable to substitution at G-6 position, as it pointed out in FT-IR analysis. Additionally, the identified dose-dependent correlation in cell viability for sulfated derivatives underscores the importance of sulfation degree in modulating cellular responses, concomitantly implicating sulfation as a causative factor in cytotoxicity [38]. However, the concentrations associated with cytotoxic effects do not align with concentration present on the surface of PLA-SF, providing further contextualization to the observed disparities in cytocompatibility. A similar result was observed for PLA-CMF. The process of carboxymethylation enhances cytocompatibility, as well as cell adhesion, growth, and the differentiation of adipose stem cells [4,27,65]. However, the interaction between the negatively charged functionalities resulting from plasma-treated surfaces and the limited number of positively charged amino groups significantly influences the immobilization of furcellaran with negatively charged groups (sulfate/carboxymethyl). This dynamic results in enhanced repulsive forces between the PLA surface and sulfated/carboxymethylated furcellaran molecules, leading to decreased binding affinity and, consequently, poorer adsorption [66]. This phenomenon manifests as the formation of localized flocculated particles on the continuous PLA surface (as indicated in SEM micrographs), which may have unpredictable biological implications. Previous studies have demonstrated that non-uniform immobilization of chondroitin sulfate onto LDPE surfaces negatively affected cell morphology [67]. To mitigate this, employing grafting through amine-based mediators can introduce additional positively charged amino groups, ensuring better stability of the immobilized polysaccharide via covalent bonding [68,69]. This approach aims to promote homogeneity in immobilization and enhance the overall biocompatibility of PLA surfaces.

The collective findings from this assay indicate that SF and CMF-functionalized PLA scaffolds exhibit no cytotoxicity towards the cells.

## 4. Conclusions

In this study, we successfully synthesized sulfated and carboxymethylated derivatives of *F. lumbricalis* polysaccharides through chemical modification, achieving DS values of 0.8 and 0.3, respectively. FT-IR and elemental analysis confirmed the successful incorporation of sulfate and carboxymethyl groups into the furcellaran backbone. The highly porous PLA scaffolds, prepared using a solution/porogen leaching method, were effectively surface-functionalized with sulfated and carboxymethylated FUR through RF air plasma treatment, as verified by XPS analysis. The survey scan XPS spectra indicated increases in characteristic elements (N and S) for SF and CMF.

Morphological analysis, conducted through SEM images, revealed the uniform distribution of polysaccharides on both the surface and within the pores of the PLA scaffolds. Notably, the surface-functionalized PLA scaffolds exhibited enhanced surface hydrophilicity and roughness compared to untreated PLA, suggesting improved biological interactions. The cytocompatibility of the prepared scaffolds was assessed using mouse embryonic fibroblast cells (NIH/3T3), and none of the samples exhibited cytotoxicity.

These results establish a theoretical foundation for the development of innovative scaffolds treated with furcellaran derivates, highlighting their potential in various biomedical applications.

## Figures and Tables

**Table 1:** Elemental composition of chemically modified furcellaran.

Sample	Elements (%*w*/*w*)				
	C	S	H	N	DS ^1^
FUR	31.5 ± 0.4	2.0 ± 0.2	4.9 ± 0.3	0.1	0.15
SF	22.8 ± 0.1	8.0 ± 0.1	4.1 ± 0.2	1	0.8
CMF	33.4 ± 0.3	0.6 ± 0.1	4.7 ± 0.2	0.1	0.3

^1^ DS—degree of substitution.

**Table 2:** Elemental surface composition of the samples.

Sample	Elements (%*w*/*w*)			
	C	N	O	S
PLA	62.8	/	37.2	/
PLA-SF	63.6	1.4	34.7	0.3
PLA-CMF	62.7	1.1	36.1	0.2

## Data Availability

Data are available from corresponding authors upon reasonable request.
